# Cellular senescence, a novel therapeutic target for mesenchymal stem cells in acute kidney injury

**DOI:** 10.1111/jcmm.16163

**Published:** 2020-12-07

**Authors:** Lingfei Zhao, Chenxia Hu, Fei Han, Dajin Chen, Yanhong Ma, Junni Wang, Jianghua Chen

**Affiliations:** ^1^ Kidney Disease Center The First Affiliated Hospital College of Medicine Zhejiang University Hangzhou China; ^2^ Key Laboratory of Kidney Disease Prevention and Control Technology, Zhejiang Province Institute of Nephrology Zhejiang University Hangzhou China; ^3^ State Key Laboratory for Diagnosis and Treatment of Infectious Diseases The First Affiliated Hospital College of Medicine Zhejiang University Hangzhou Zhejiang China

**Keywords:** acute kidney injury, cellular senescence, mesenchymal stem cells

## Abstract

Cellular senescence is a widespread cellular programme that is characterized by permanent cell cycle arrest. Senescent cells adopt a changed secretory phenotype that can alter cellular function. For years, cellular senescence has been thought to be a protective factor against cancer; however, it is now recognized that it has a dual effect on individuals. Co‐ordinated activation of cellular senescence provides advantages during embryogenesis, wound healing, tissue repair and inhibition of tumorigenesis. On the other hand, the aberrant generation and accumulation of abnormal senescent cells lead to the development of age‐related conditions and tissue deterioration. During acute kidney injury (AKI), the kidney faces multiple types of stressors and challenges, which can easily drive cellular senescence. How to appropriately progress through the cell cycle and minimize long‐term damage is of great importance to the acquisition of adaptive repair considering that no available therapeutic interventions can reliably limit injury, speedy recovery or improve the prognosis of this syndrome. Whether the manipulation of cellular senescence can become a novel therapeutic target in AKI and reignite clinical and research interest remains to be determined. Here, we share our current understanding of the role of cellular senescence in AKI, along with examples of the application of mesenchymal stem cells (MSCs) for targeting this disorder during its treatment.

## 1 INTRODUCTION

Acute kidney injury (AKI) is a common clinical syndrome with multiple risk factors and causes. Pathophysiologically, it is characterized by a sudden decrease in glomerular filtration rate and deterioration in kidney function, accompanied by a consequent disruption in fluid, electrolyte, acid‐base and metabolic homoeostasis. Clinically, it can range from small changes in some biomarker levels without overt manifestation to serious clinical consequences in some circumstances.^1^ Currently, most studies follow the Kidney Disease Improving Global Outcomes diagnosis criteria and staging of AKI in adult patients, which is based on the level of serum creatinine, with or without the manifestation of urine output alterations.^2^ Epidemiologically, despite the great progress in modern medicine, AKI is still regarded as a major global public health concern, affecting approximately 25% of hospitalized patients and 13 million people per year.^3^, ^4^

Traditionally, it has been accepted that the kidney has a strong regenerative capacity against acute injury. Unless a fatal insult occurs, a mild or moderate episode of AKI leaves no sequelae in its survivors. However, an increasing number of epidemiological studies indicate a strong link between an episode of AKI and poor outcomes. The maladaptive repair after AKI can lead to continuous deterioration in renal structure and function and ultimately progress to chronic kidney disease (CKD), which is termed AKI‐CKD transition. In 2019, a meta‐analysis that included 82 studies, compromising over 2 million patients, indicated that the risk of developing CKD and end‐stage renal disease in patients who underwent AKI was 2.67‐ and 4.81‐folds, respectively, higher than individuals without a history of AKI.^5^ Furthermore, temporary AKI resulting from cardiac surgery or contrast toxicity would increase the mortality rate in the long term.^6^, ^7^

Particularly, emerging evidence indicates that AKI shares some common features with cellular senescence, a diverse cellular programme in eukaryotes. Cellular senescence is thought to participate in acute tissue repair, such as wound healing. However, if abnormally activated, it leads to reduced proliferative capacity, persistent inflammation and formation of fibrosis, similar to the pathophysiological changes during maladaptive repair after AKI, indicating that the reduced regenerative capacity after AKI‐induced cell injury was associated with the uncontrolled cellular senescence machinery.^8^, ^9^, ^10^ In the context of AKI, registry data have also demonstrated that the incidence of AKI was much higher in CKD patients and healthy elderly population, who presented with a high background level of cellular senescence.^11^, ^12^ These examples implicate a proof‐of‐concept relationship between post‐insult cellular senescence and a poor fibrotic outcome of AKI. Complemented by a recent finding that selective elimination of senescent cells in mice significantly extended their healthy lifespan and alleviated physical dysfunction, it could be assumed that cellular senescence could become a novel target for the treatment of AKI, and re‐balancing its homoeostasis has potential benefits.^13^

Despite great advances in the understanding of the complex pathophysiologic alterations during AKI, current treatment is still confined to supportive strategies such as dialysis. Promising trials of agents, including diuretics, dopamine and atrial natriuretic peptide, were effective in animal experiments but failed when translated into clinical settings.^2^, ^14^, ^15^ Lack of a valid intervention to stop the progression of this syndrome and associated systemic maladies have resulted in a poor prognosis. It is estimated that the mortality rate of AKI ranges from 50% to 70% in intensive care unit patients.^16^ With its high incidence, which is a heavy economic burden on the healthcare system, over $10 billion is spent annually on it in the United States,^1^ exploring novel treatments is urgently needed.

In the last decade, mesenchymal stem cells (MSCs) have been extensively studied in the context of AKI both in experimental models and clinical trials. In animal studies, MSCs are effective in covering most of the aetiologies of AKI, including ischaemia/reperfusion (I/R) injury,^17^ toxicants ^18^ and sepsis.^19^ In the clinical settings, a phase 1 clinical trial (NCT00733876), which involved 18 patients who were at high risk of developing AKI following cardiac surgery, showed that MSCs were protective against AKI development.^20^ One of the advantages of MSC‐based therapy, as compared with pharmacologic interventions, was that it showed its effectiveness by targeting multiple pathways that are known to be activated in the pathophysiological process during AKI. By alleviation of inflammation, apoptosis, acceleration of angiogenesis and modulation of the immune reaction, MSCs contribute to limiting injury and promoting regeneration.^21^

In addition to the above‐mentioned traditional views, a study hinted that MSCs could also alleviate renal cellular senescence.^22^ Given this scenario, the main question is whether the regulation of cellular senescence can be regarded as a novel target for MSC‐induced protection against AKI? In this review, we have included all available studies that explored the application of MSC‐based therapy for targeting cellular senescence during AKI. The current understanding of the mechanism of senescence in AKI is examined, and the potential therapeutic effect of using MSCs to target senescent cells during this phase is discussed. By summarizing the available studies, we intend to provide an up‐to‐date review to support the role of anti‐cellular senescence effects of MSCs on AKI and aim to highlight the potential future research and translational relevance.

## 2 WHAT IS CELLULAR SENESCENCE

Originally, senescence was used by biologists to describe the decline in the function of an organism with time and was thought to be close to the term ‘ageing’ for a long time. In the 1960s, Leonard Hayflick first systematically described this word, which referred to the phenomenon where human fibroblasts permanently arrest cell division after prolonged cell culture in vitro for telomere shortening.^23^, ^24^ Nowadays, however, cellular senescence is characterized by a permanent fate change in injured cells. In addition to ageing‐related disorders, the senescence programme is also implicated in a diverse range of biological processes, including embryogenesis, wound healing, tumorigenesis and tissue regeneration.^25^, ^26^, ^27^, ^28^, ^29^ Unlike apoptosis, which results in a static end‐point of the injured cells, cellular senescence is a complex cellular process that converts cells into irreversible growth arrest. Although most senescent cells may remain viable and metabolically active, they inevitably adopt an altered phenotype, including changes in chromatin, gene expression and protein secretion, which is termed senescence‐associated secretory phenotype (SASP).^25^ The SASP factors contain a large number of pro‐inflammatory cytokines, chemokines, growth factors and matrix‐degrading proteases, such as transforming growth factor‐β1 (TGF‐β1), interleukin‐1 (IL‐1), IL‐6, IL‐8, WNT16B, plasminogen activator inhibitor 1 (PAI‐1) and monocyte chemoattractant protein 1(MCP‐1).^30^, ^31^, ^32^, ^33^, ^34^ Through a paracrine and autocrine fashion, these factors hold the ability to maintain or exacerbate the status of senescence within tissues.^35^ Data from the study by Xu et al demonstrated that cellular senescence could be spread between individuals through transplantation of senescent cells, indicating the participation of paracrine senescence.^13^ In the following section, we will discuss the definition, mechanisms and functions of cellular senescence.

### 2.1 Definition of cellular senescence

Although the physical and functional alterations in cellular senescence in vitro are well described, unfortunately, there is no universally accepted definition of cellular senescence in vivo. Some features are summarized as potential markers of cellular senescence. Staining of senescence‐associated β‐galactosidase (SA‐β‐gal), increased expression of cyclin‐dependent kinase (CDK) inhibitors, including p21^cip1^, p16^ink4a^, and p19^arf^, confirmation of SASP, and the absence of coexisting cell proliferation are regarded as important markers for cellular senescence, which can also be called multiple semi‐selective senescent cell characteristics, as proposed by the Van Deursen group.^25^

### 2.2 Signal pathways for cellular senescence

**FIGURE 1:**
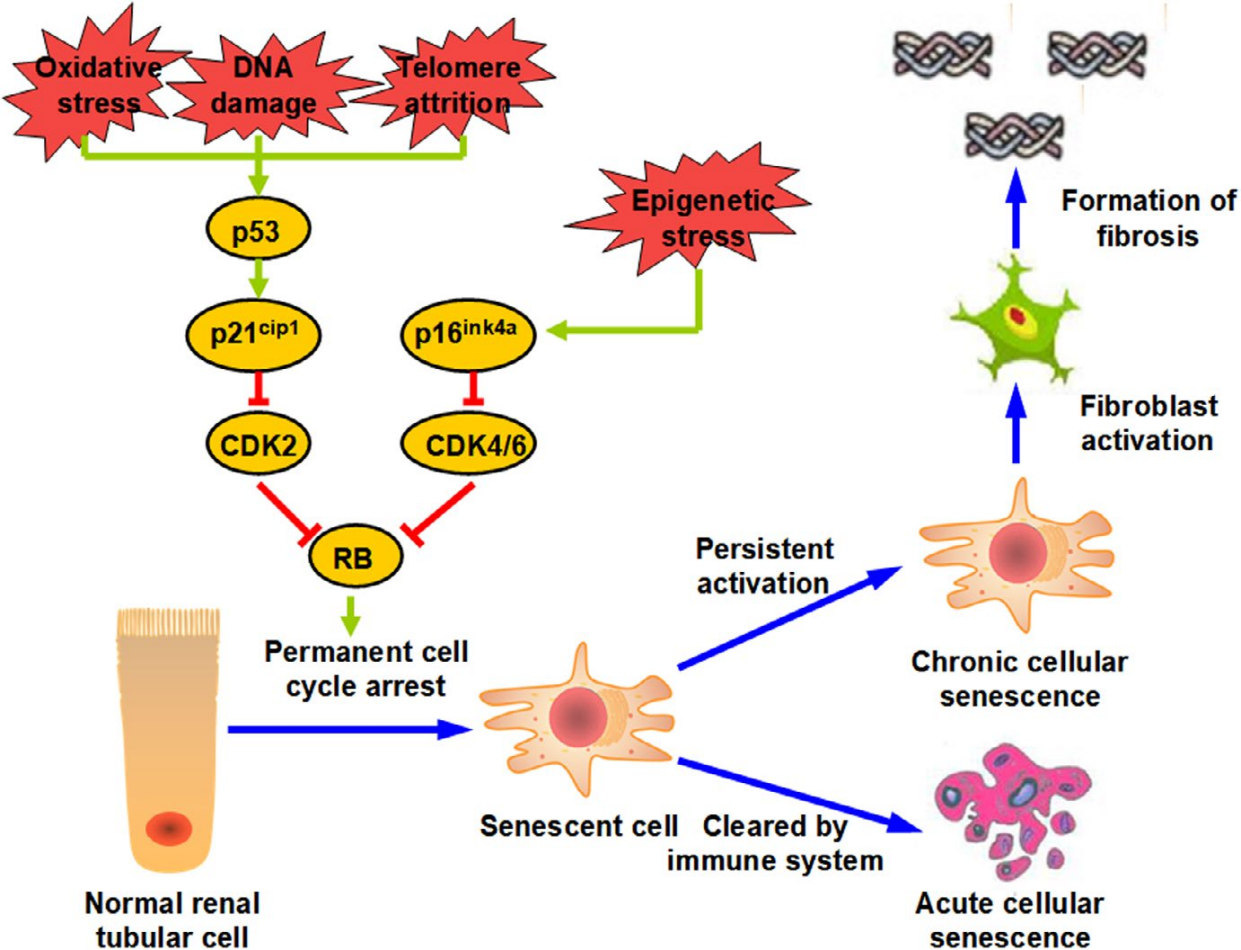
Signal pathways to the development of cellular senescence in I/R‐AKI. During AKI, a variety of cellular insults act as distinct or combined factors to engage in the cellular senescence programme and induce cell arrest at permanent cell cycle (left side). Cellular senescence, which is scheduled and cleared by the immune system in the short term, will leave no sequelae, termed acute cellular senescence. However, if persistently activated, it will lead to chronic cellular senescence, activating fibroblast and eventually resulting in the formation of fibrosis (right side) (see text for more details). I/R, AKI, ischaemia/reperfusion‐induced acute kidney injury

As mentioned above, the initial cellular senescence occurs in various cellular signal cascades in various types of physiological and pathological processes. However, despite the abundance of cell‐intrinsic and cell‐extrinsic stimuli, current insights have shown that most of them are present in at least active two well‐investigated pathways, which are the p53/p21^cip1^ and p16^ink4a^ pathways or both (Figure 1). The activation of the p53/p21^cip1^ pathway can be induced by stressors such as oxidative stress, telomere attrition and DNA‐damage response.^36^ Alternatively, p16^ink4a^ is mainly induced by oncogene inactivation and mitotic or epigenetic stressors.^37^ The increased p53/p21^cip1^ transcription enhances the checkpoint activity by targeting CDK2, whereas p16^ink4a^ does so by targeting CDK4 and CDK6 complexes. Both pathways eventually inactivate the retinoblastoma protein, resulting in cell cycle arrest.^38^, ^39^

Although great advances have been made in the detailed insights into the mechanisms applied to cellular senescence, it is necessary to point out that current research is largely based on ex vivo cell culture experiments. The mechanisms involved in the regulation of in vivo pathology are not well understood. Ex vivo research on cellular senescence is always conducted under a single stimulus, for example high‐dose radiation, drugs or oncogenes. However, the actual in vivo process of cellular senescence is far more complex. Under pathological status, individuals face multiple cellular pressures. Taking I/R‐induced AKI (I/R‐AKI) as an example (Figure 1), however not unexpectedly, mitochondrial dysfunction preceded the physiopathology following the insult and contributed to the main source of excess reactive oxygen species (ROS).^40^ Oxidative stress, on the one hand, can directly induce cellular senescence by triggering signalling through the p53/p21^cip1^ pathway. On the other hand, it can cause DNA damage and subsequently halt cell cycle progression.^41^, ^42^ In addition to replication‐induced telomere attrition, stress‐induced telomere shortening was also observed after AKI, which could be another induction of cellular senescence.^43^ In addition to the activation of the p53/p21^cip1^ pathway, recent studies have highlighted that modifications in epigenetic levels also determine the outcome of AKI.^44^, ^45^ These findings suggest that cellular senescence during AKI can also be derived from epigenetic stress through the p16^ink4a^ pathway. Advanced studies that combined diverse senescence‐promoting stressors, including cell‐intrinsic and cell‐extrinsic factors, will expand our understanding of this vital but complicated process in the future.

### 2.3 Acute and chronic cellular senescence

We can now conclude that there are some basic characteristics of cellular senescence. As we mainly focus on AKI in this manuscript, it is widely accepted that cellular senescence will be activated after an acute insult. The next question is as follows: how about its role in this process?

Cellular senescence activated post‐injury is thought to have time‐dependent contributions to tissue regeneration. Based on the specific time point, it can be classified into acute cellular senescence and chronic cellular senescence.

Acute cellular senescence is thought to be a part of tightly orchestrated biological processes. It is always triggered by a single specific stimulus and is quickly removed by the immune system as programmed.^36^ Based on these characteristics, acute cellular senescence acts as part of the healing in the success of tissue repair. After a cutaneous wound insult, senescent cells appeared early and induced myofibroblast differentiation through the secretion of platelet‐derived growth factor AA (PDGF‐AA), which could promote wound closure.^46^ Similar to skin repair, in a murine acute liver injury model, senescent‐activated hepatic stellate cells following injury helped in the reduction in extracellular matrix component secretion, promotion of extracellular matrix‐degrading enzyme synthesis and the amelioration of liver fibrosis.^47^

In contrast, probably by exposure to combined effects or under distinct, strong, or repeated stimuli, cellular senescence will become unscheduled and will switch from temporal to persistent cell cycle arrest, called chronic cellular senescence. Therefore, it is easy to understand that, in contrast with the positive role of acute cellular senescence, the induction of chronic cellular senescence after an injury is usually harmful and contributes to the development of a chronic disease rather than be a bystander. The accumulation of senescent cells and continued secretion of SASP factors over time disrupt the stem‐cell niche, perturb tissue organization, induce epithelial‐mesenchymal transition (EMT), eventually shorten lifespan and promote tissue deterioration in various organs, including the muscle, adipose tissue, heart and kidneys.^31^, ^48^, ^49^, ^50^ Conversely, elimination of senescent cells in murine models has been shown to prevent or delay the onset of age‐related diseases and enhance the health span.^13^, ^51^ These findings provide credible evidence that suggests a dual role for cellular senescence in tissue injury, with early beneficial effects followed by deleterious consequences in the long term. Whether cellular senescence plays a role during AKI, especially in the repair process after AKI, is unknown. In the last decade, great interest has been generated in this area and we intend to discuss this issue in the following section.

## 3 CELLULAR SENESCENCE AND AKI

The fact that insufficient renal repair after an episode of AKI in elderly patients might be caused by the accumulation of senescent cells was first raised almost 20 years ago, indicating a role for cellular senescence in AKI.^52^, ^53^ Until recently, findings from gain and loss studies revealed a valid relationship between cell senescence and AKI. During AKI, cellular senescence can be occurred both in the cortex and medulla of renal tissues. Despite tubular epithelial cells which are the majority of senescent cells, cells including podocytes, vascular smooth muscle cells, endothelial cells, interstitial cells, mesangial cells and immune cells can also become senescent.^54^ It is known that regeneration after AKI largely relies on the surviving mature cells, rather than on intrinsic renal stem cells. In response to the rapid cell death following injury, it was estimated that approximately 70% of surviving, normally quiescent tubule cells could re‐enter the cell cycle within the first 24 h, promoting renal regeneration.^55^, ^56^ However, a prolonged entrapment in cell cycle arrest after injury will affect the proliferation of renal cells, delay the recovery of renal function and give rise to the development of CKD. It seems that cellular senescence may cause trouble in the regeneration of AKI.

Recent findings have shown that after renal I/R injury, the levels of p21^cip1^, p53 and p16^ink4a^ increased, which indicates the acceleration of cell senescence.^57^ Data from a study conducted by Yang et al also demonstrated that acute injury factors, including ischaemia, toxicants and obstruction, could induce proximal tubular cell arrest at the G2/M phase. These cells could further activate c‐jun NH(2)‐terminal kinase signalling, acting as a pro‐fibrogenetic factor in the injured kidney.^41^ Similarly, stathmin is an 18‐kDa phosphoprotein that is involved in the formation of functional mitotic spindles and cell division.^58^ Stathmin deficiency in mice suffering I/R‐AKI was thought to be a negative factor in the regulation of G2/M transition and the exacerbation of renal injury.^59^ In contrast, the inhibition of some senescent factors, such as ablation of p16^ink4a^ or blockage of p21^cip1^ before AKI, displayed a better outcome compared with the control group after injury.^60^, ^61^ Similarly, the administration of a p53 inhibitor to bypass G2/M arrest, a cellular senescence event, also rescued fibrosis in a unilateral I/R‐AKI model.^41^ These examples complemented the results of epidemiological studies concluding that AKI produces SASP after injury, gradually leading to the accumulation of renal fibrosis and the development of CKD.

However, it is also important to note that there are reports that have demonstrated that strategies using induction of cell cycle arrest were conversely protective against AKI both in vitro and in vivo. Contrary to the traditional view that the induction of cellular senescence by p21^cip1^ after AKI is detrimental to the following repair, Megyesi et al concluded that p21(‐/‐) mice were more susceptible to I/R‐AKI, with parallel inappropriate elevated cell cycle activity, indicating the beneficial role of cellular senescence in kidney regeneration.^62^ Similarly, mice treated with a specific CDK4/6 inhibitor, PD 0332991, before I/R‐AKI, exhibited significant amelioration of renal injury. The pharmacological quiescence of renal proximal tubule cells by stopping re‐entry into the cell cycle through the blockage of the CDK4/6 pathway contributed to better renal function after AKI.^63^, ^64^

These inconsistencies described a more complicated picture of the role of cellular senescence in AKI. In our opinion, this is the duration of cellular senescence after AKI. As mentioned above, cellular senescence can be divided into acute and chronic cellular senescence, both of which have distinct roles in the process of tissue repair, mainly based on a time‐dependent principle. The duration of acute cellular senescence is short and programmed. Short‐time arrest in the cell cycle contributes to avoiding uncontrolled mitosis and providing more time for DNA repair. After the acute phase, acute senescent cells are scheduled to be eliminated by the immune system, leaving no effect on long‐term renal function. However, chronic cellular senescence lasts longer and is more chaotic than acute cellular senescence. The long‐term activation of cellular senescence turns cells into permanent growth arrest, presented as SASP. This undesirable process, with no surprise, is associated with progressive interstitial fibrosis and detrimental long‐term consequences.

In the process of AKI repair, the situation is not different. After an AKI insult, in most instances (a single mild or moderate hit), due to the strong compensatory capacity of the kidney, the kidney can experience adaptive repair and leave no sequelae in the future. Alternatively, if a single hit is severe or the kidney undergoes repeated episodes of AKI during a relatively short period, multiple pathophysiological events occur, abnormal tubular regeneration is triggered and the progression to CKD fosters.^65^ The different outcomes may be caused by the different types of cellular senescence that developed after AKI. Maladaptive repair is thought to account for the activation of chronic cellular senescence. Yang et al demonstrated that the degree of damage is a major determinant of the development of chronic cellular senescence after AKI. After mild kidney injury, such as a moderate or reversible I/R‐AKI, the increased numbers of cells arrested in the G2/M phase only existed from days 1‐5, and these mice did not present with renal interstitial fibrosis in the long term. In contrast, if the injury is severe, such as suffering from severe I/R‐AKI or a nephrotoxic hit, G2/M‐arrested cells persisted for as long as 42 days and would induce significant renal interstitial fibrosis.^41^ The degree of decline in Klotho expression, an anti‐senescence protein, was also dependent on the duration of ischaemia.^66^ Venkatachalam et al tried to explain these facts from the perspective of pathology. They pointed out that, by itself, one focus of the renal fibrotic area after injury was not a progressive factor, but a self‐limiting repair process that could restrict injury sharply demarcated from recovered or normal tissues. The progression to CKD requires additional factors, such as capillary rarefaction, mitochondrial injury and persistent inflammation, all of which could be ascribed to the abnormal activation of chronic cellular senescence.^67^

## 4 BENEFICIAL EFFECTS OF MSC‐BASED THERAPY FOR TARGETING CELLULAR SENESCENCE DURING AKI

Considering the above‐mentioned points, enhancing the long‐term therapeutic effects of AKI and preventing the development of AKI‐CKD transition by reducing the incidence of the chronic senescent phenotype in the kidneys after AKI are meaningful. In recent years, agents termed senolytics have been developed, which can clear away senescent cells. Among them, MSCs are promising candidates (Table 1).

**TABLE 1:** Associated articles demonstrating the beneficial effects of MSC‐based therapy for targeting cellular senescence during AKI

Year	Animal	AKI model	MSC source	Outcomes	References
2016	Rats	Sepsis	WJ‐MSCs	↑Klotho;↓Pathological score; ↑Renal function	Condor (73)
2018	Mice	I/R	BM‐MSCs modified with Klotho	↑Klotho; ↓Wnt/β‐catenin pathway; ↓Renal atrophy; ↓Pathological score; ↑Renal function	Zhang (74)
2019	Rats	I/R	BM‐MSCs modified with Klotho	↑Klotho; ↑FoxO1/p‐FoxO1; ↑SOD; ↓MDA; ↓Pathological score; ↑Renal function	Xie (78)
2017	Rats	I/R	UC‐MSCs	↑Klotho; ↓β‐gal; ↓ p21^cip1^, p16^ink4a^; ↓TGF‐β1; ↓Senescence‐related proteins and microRNAs; ↓Pathological score; ↑Renal function	Rodrigues (80)
2017	Mice	Glycerol	100K‐EVs derived from BM‐MSCs	↑mRNAs of CCNB1, CDK8, CDC6; ↑Proliferation; ↓Pathological score; ↑Renal function	Bruno (81)

Abbreviations: AKI, acute kidney injury; BM‐MSCs, Bone marrow‐derived MSCs; CCNB1, cyclin B1; CDC6, cell division cycle 6; CDK8, cyclin‐dependent protein kinase 8; EV, Extracellular vesicles; I/R, ischaemia/reperfusion; MDA, malondialdehyde; MSCs, mesenchymal stem cells; SOD, superoxide dismutase; TGF‐β1, transforming growth factor‐β1; UC‐MSCs, Umbilical cord‐derived MSCs; WJ‐MSCs, Wharton's jelly‐derived MSCs.

Until now, most studies exploring the application of MSC‐based therapy for targeting cellular senescence during AKI were designed with reference to Klotho. Although not included in the classical cellular senescence signal pathway, Klotho has been predominantly regarded as an anti‐ageing factor for a long time. Klotho is a transmembrane protein that is mainly expressed in renal tubular epithelial cells (TECs). The deficiency of the Klotho gene in mice resulted in various ageing‐associated features, whereas overexpression of Klotho extended their lifespan.^68^, ^69^ In terms of mechanism, recent studies have found that the anti‐ageing effect of Klotho was closely related to cellular senescence, specifically achieved via mitogen‐activated kinase or the alleviation of oxidative stress.^70^, ^71^ When suffering AKI, mice with defects in Klotho presented more severe functional and histological damage compared with the wild type, whereas those supplemented with recombinant Klotho showed fewer alterations.^72^ These findings revealed that Klotho could serve as a potential therapeutic target for AKI, and its renoprotective effects are associated with cellular senescence.

Condor et al demonstrated the effects of targeting cellular senescence during MSC‐based therapy against AKI.^73^ Intraperitoneal injection of the human Wharton's jelly‐derived MSCs into rats that underwent caecal ligation and puncture successfully increased renal Klotho expression and attenuated AKI. This evidence indicates that the partial restoration of Klotho levels by MSC intervention was potentially beneficial for AKI treatment. In addition to sepsis‐induced AKI, I/R‐AKI is a more common type of AKI in clinical settings. Zhang et al attempted to transplant bone marrow‐derived MSCs (BM‐MSCs) modified by the Klotho gene into mice suffering from I/R‐AKI.^74^ Less renal atrophy, decreased pathological score and better renal function were observed, demonstrating that the overexpression of Klotho in BM‐MSCs was associated with an increased renal protective effect against AKI. To verify the hypothesis that the beneficial effect during this process was due to the Klotho protein secreted from MSCs, the authors measured the value of Klotho and β‐catenin in TECs in an in vitro model of I/R injury. Klotho deficiency activated the Wnt/β‐catenin signalling pathway, which further arrested cells at the G2/M phase, inducing cellular senescence.^75^, ^76^ Consistent with previous results, they confirmed that the expression of Klotho in TECs suffering I/R injury recovered after incubation with Klotho gene‐modified BM‐MSCs, together with a dramatic suppression of β‐catenin, confirming their hypothesis. Similarly, mammalian members of the FoxO class are important regulators of oxidative stress.^77^ A study by Xie et al concluded that by their involvement in the regulation of Klotho/FoxO1 axis and participation in the inhibition of downstream cellular oxidative stress, Klotho‐modified BM‐MSCs provided more renal protection than that provided to a group that received normal BM‐MSCs therapy.^78^

It is well recognized that AKI is a risk factor for the development of CKD.^79^ To assess the anti‐cellular senescence effects of MSCs for the treatment of AKI in the long term, Rodrigues et al transplanted umbilical cord‐derived MSCs into a rat model of I/R‐AKI.^80^ Although on day 7, the serum creatinine and urea levels were comparable in both groups, UC‐MSC treatment resulted in a quicker recovery of renal function, in parallel with the normalization of senescence‐related markers, including Klotho, p21^cip1^, p16^ink4a^, TGF‐β1 and β‐gal. On day 49, however, the value of serum creatinine, urea and FENa was all significantly higher in the untreated rats than in the UC‐MSC‐treated rats, indicating the development of CKD in the control group. Moreover, the pathological scores and the expression of Klotho and β‐gal were also in line with the change in renal function. These examples suggest that the anti‐cellular senescence effects of MSCs not only helped in the amelioration of renal function loss in the acute phase but also protected against a maladaptive repair in the long term.

Except for the regulation of Klotho, one study suggested that there are other mechanisms underlying MSC‐based therapy targeting cellular senescence during AKI. It is known that different extracellular vesicles (EVs) of MSCs have different biological activities because of their heterogeneity. Bruno et al separately collected 10K and 100K population of EVs derived from MSCs using ultracentrifugation, and found that only the 100K population showed positive therapeutic effects, whereas the 10K population was ineffective. After conducting molecular content analysis, they found that specific cell cycle mRNAs (cyclin B1 (CCNB1), CDK8 and cell division cycle 6 (CDC6)) were selectively enriched in the 100K population. This fact could explain the distinct therapeutic effects of different populations of MSCs against AKI. CCNB1, CDK8 and CDC6 are all important cell cycle regulators that influence cell cycle entry and progression. The abundant existence of these mRNAs in the 100K population guarantees a smooth cell division cycle, enhances cell proliferation and helps ameliorate renal dysfunction.^81^

### 4.1 BENEFICIAL EFFECTS OF MSC‐BASED THERAPY FOR TARGETING CELLULAR SENESCENCE IN TRANSPLANTED MSCS DURING AKI

Despite targeting cell senescence in the kidney, cell senescence in transplanted MSCs is of equal importance and should be investigated. It is well known that paracrine actions rather than cell differentiation is the major mechanism that underlies the therapeutic efficacy of MSCs.^82^ The paracrine function of MSCs could be tightly regulated by the RAP1/NFkb signalling pathway.^83^ However, ageing MSCs revealed as SASP with low expression of several cytokine and chemokine receptors, which were crucial for their migration and anti‐inflammatory response, compromising their protective roles in acute lung injury.^84^ Another example in this case was that bone marrow‐derived stem cells from young mice could attenuate cell senescence in old mice, whereas stem cells from old donors were less effective.^22^ These findings suggest that the therapeutic effects of MSCs may depend on the status of cellular senescence in MSCs. However, in the setting of MSC‐based therapy against AKI, the matter of cell senescence in transplanted MSCs is much more common than the traditional assumptions.

First, compared with allogenic MSC transplantation, autologous therapy has the unique advantage of overcoming immune rejection.^85^ However, AKI is easier to develop in the elderly than in young people. The viability and functionality of MSCs harvested from the elderly conceivably could be attenuated by their increased age and level of cellular senescence.^86^ Second, under in vitro expansion conditions, MSCs show a tendency towards cellular senescence. Ex vivo culture environments would cause MSCs to gradually lose their multiple differentiation potential and decrease their proliferation rate, thereby reducing their bone‐forming efficiency in vivo.^87^ Lastly, after being transplanted, MSCs meet a harsh microenvironment in vivo. Uraemic toxins circulating in patients with AKI are known to cause cellular senescence in MSCs, which diminishes their proliferative capacity and further affects their therapeutic potential.^88^ In conclusion, numerous studies have suggested that MSC senescence is vital for the clinical application of MSC‐based therapy against AKI, and studies exploring therapeutic approaches to target MSC senescence are required.

Until now, only one study has demonstrated improved efficiency in the reversal of AKI by targeting senescence in MSCs.^74^ The overexpression of the Klotho gene in MSCs effectively increased their proliferative ability both in vitro and in vivo, which further helped contribute to better therapeutic effects in I/R‐AKI rats. The mRNA levels of octamer‐binding transcription factor 4 (OCT4) and Nanog were also dramatically higher in Klotho gene‐modified BMSCs than in the control group, suggesting that the elevated proliferative capacity was due to the regulation of pluripotency in MSCs by an anti‐cellular senescence strategy.

Although not related with AKI, data from myocardial infarction and cerebral ischaemia also provide us useful information about the ejuvenation of ageing MSC in improving its clinical efficacy. Liang et al tried to genetically modify ageing MSCs with Erb‐B2 receptor tyrosine kinase 4 ( ERBB4), which was a essential pathway in regulating ageing. The up‐regulation expression of ERBB4 in ageing MSCs significantly ameliorated its senescent phenotype in vitro and helped enhance its therapeutic effects in vivo.^89^ Similarly, Zhang et al concluded that hypoxia conditioning could also improve the neuroprotection effects of ageing MSCs.^90^

## 5 FUTURE DIRECTIONS

We summarized the beneficial effects of targeting cellular senescence in kidney cells as well as in MSCs during the protection of MSC‐based therapy against AKI. Despite its promising future, some questions still need to be addressed before clinical translation.

The first question is the identification of senescent cells. Accurate detection of senescent cells in vivo is of paramount importance, especially considering strategies such as senolysis. Identifying the specific differences in senescent cells and targeting them just like those used to kill cancer cells will largely affect the efficacy of senolysis and avoid potential off‐target effects.

Although a consensus that combines both particular phenotypes and signal pathways of cellular senescence has been attained in the field currently, it is important to note that none of them is specific to the characteristics of this programme. The presence of SA‐β‐gal can also be detected in macrophage subpopulations or osteoclasts,^91^, ^92^ and p19arf and p16ink4a are not specifically unique to senescent cells, as they are expressed in immune cells as well.^93^ Several reasons may explain this difficulty. First, cellular senescence is diverse. It can be activated in a broad spectrum of cells, tissues and organs, in different physiological and pathophysiological processes induced by various stressors. Second, cellular senescence is complex. Despite great progress in the understanding of cellular senescence in the last few years, the detailed molecular mechanism of cellular senescence is still not well understood. Third, cellular senescence is heterogeneous. Even in the same tissue, senescent cells are constantly evolving. These drawbacks make it challenging to specifically identify and characterize these cells, especially in vivo. Recent advances in single‐cell sequencing should facilitate this situation.

The second question concerns the time‐point to intervene. As mentioned above, the duration of cellular senescence is a key factor for its roles during AKI, with benefits in the short term but will be outweighed if prolonged. Unfortunately, it is still difficult to differentiate between acute cellular senescence and the chronic one. However, none of the available studies investigated the delicate balance when applying MSC‐based therapy targeting cellular senescence during AKI. All of them chose to transplant MSCs either immediately after an injury or a few hours later. The transplanted MSCs showed positive outcomes in the attenuation of cellular senescence and alleviation of renal injury, indicating that senescent cell accumulation at this time point is primarily detrimental. Further research with the help of a model system that allows different types of senescent cells to be specifically regulated is anticipated.

Third, the route of MSC delivery should be taken into consideration. All available studies chose to intravenously inject transplanted MSCs. Through paracrine action, MSCs exert their impact systematically, especially in the lungs and spleen, where most of the transplanted MSCs are tapped in. However, there are complex and diverse roles of senescence in different biological processes in various tissues. In particular, evidence suggests that psychological activity cellular senescence in some ways is beneficial for tissue development, repair and cancer defence.^36^ Will the beneficial effect of MSCs targeting cellular senescence during AKI be at the expense of deterioration in other organ functions? Will local transplantation be safer? Despite proper timing, the best delivery route should also be carefully considered.

Fourth, the relationship between MSCs, cellular senescence, and the immune system is still under debate. The immune system is believed to be the major participant in the clearance of senescent cells. Dysfunction in the immune system coincides with the accumulation of chronic senescent cells, suggesting the evasion of immune clearance.^94^ Meanwhile, there is a great body of evidence showing that MSCs are also well recognized as immunoregulators. Whether the potential for MSC therapy targeting AKI senescence is the direct function to modulate cellular senescence or the reflection of improved immune system ageing is yet to be determined? Until now, no study has focused on this field, which merits further exploration.

Fifth, the available studies included in our review did not cover induced pluripotent stem‐cell‐derived mesenchymal stem cells (iPSC‐MSCs). Compared with MSCs which was derived from usual routes, including BM, Wharton's jelly, fat tissues and umbilical cord, iPSC‐MSCs could overcome some conventional drawbacks, such as scarce cell source, limited expansion potential and cellular senescence.^95^ Some studies have reported that the therapeutic potential of iPSC‐MSCs was superior over others in hindlimb ischaemia, myocardial infarction and vascularized composite allotransplantation.^96^, ^97^, ^98^ However, one concern with iPSC‐MSCs in clinical settings is its tumorigenicity. Recently, a phase I clinical trial which applied good manufacturing practice‐grade iPSC‐MSCs to treat acute steroid‐resistant graft‐vs.‐host disease has shown promising outcomes.^99^ More researches in the future may provide us a clearer understanding in this field.

Finally, will the depletion of senescent cells by MSCs increase cancer incidence in vivo? Cellular senescence is thought to play a role in cancer defence. Decreased expression of senescence markers is associated with poor prognosis in human renal cell carcinoma.^100^ Inducing cell cycle arrest in the process of DNA repair during environmental stresses is also fundamental to correct cell proliferation. Meanwhile, the concern of tumorigenicity by MSCs is also raised from time to time. However, a recent study suggested that the benefits originating from targeting senescence do not increase the risk of tumour development, indicating the difference between the depletion of accumulated chronic senescent cells and those deleted in response to DNA damage in the acute phase.^31^ In the context of MSCs, until now, no studies have reported de novo tumour formation in vivo following MSC injection in humans. The available evidence is sufficient to guarantee the safety of MSC application in targeting cellular senescence in vivo.

## 6 CONCLUSIONS

In conclusion, cellular senescence is a common pathophysiological process during AKI and is related to its outcome. Targeting cellular senescence by MSC‐based therapy no doubt showed its beneficial effects against AKI both in kidney cells and MSCs. However, some important questions remain to be answered before translation to clinical settings. In the next few years, we are expecting to see an explosion of research exploring details in the identification, mechanism, and functions of cellular senescence in vivo, and the application of this information to promote the long‐term prognosis of AKI.

## CONFLICT OF INTEREST

The authors confirm that there are no conflicts of interest.

## AUTHOR CONTRIBUTION

**Lingfei Zhao:** Conceptualization (equal); Writing‐original draft (equal); Writing‐review & editing (equal). **Chenxia Hu:** Funding acquisition (equal); Writing‐original draft (equal); Writing‐review & editing (equal). **Fei Han:** Writing‐review & editing (equal). **Dajin Chen:** Writing‐review & editing (equal). **Yanhong Ma:** Writing‐review & editing (equal). **Junni Wang:** Writing‐review & editing (equal). **Jianghua Chen:** Conceptualization (equal); Funding acquisition (equal).

## Data Availability

Not applicable.
